# Safe Patient Handling Musculoskeletal Injury-Prevention Smartphone App for Community Health Care Workers: Mixed Methods Feasibility Study

**DOI:** 10.2196/70681

**Published:** 2025-10-31

**Authors:** Alfred S Y Lee, Sarah Burrell, Sandy Courtnall, Shelly Wake, Ryan E Rhodes

**Affiliations:** 1 Behavioural Medicine Laboratory School of Exercise Science, Physical and Health Education University of Victoria Victoria, BC Canada; 2 Island Health Victoria, BC Canada

**Keywords:** ergonomics, mHealth, occupational safety, user-centered design, workplace injuries

## Abstract

**Background:**

Safe patient handling is critical for reducing musculoskeletal injuries among health care workers; yet, community health care workers often face barriers such as limited access to training and real-time resources.

**Objective:**

This study had three objectives: (1) provide detailed insights into the unmet needs of Island Health community health care workers with respect to safe patient handling resources and access to information, (2) translate those needs into a user-centered prototype of the Safe Patient Handling Musculoskeletal Injury-Prevention (SPH MSIP) smartphone app through an iterative co-design process, and (3) establish the acceptability and feasibility of SPH MSIP app to support community health care workers’ safe patient handling practices using a mixed methods design.

**Methods:**

A 3-phase participatory study was conducted. Phase 1 identified unmet safe patient handling needs through participatory meetings with 6 community health care workers, aligning with objective 1. Phase 2 involved developing the SPH MSIP app using co-design methods, integrating user feedback to address challenges such as guidance for high-risk tasks and intuitive design, addressing objective 2. Phase 3 evaluated the app’s feasibility and acceptability, aligning with objective 3. The study recruited 28 participants who used the app for one month. A single-group mixed methods design was used, incorporating quantitative metrics such as recruitment (≥50%), retention (≥75%), and satisfaction (mean score ≥4). Qualitative feedback was gathered through small-group interviews to understand usability, usefulness, and integration into workflows.

**Results:**

In phase 1, community health care workers identified barriers, including limited safe patient handling, refresher training, and isolation during tasks. In phase 2, the app was developed to address these safe patient handling needs, incorporating features like scenario-specific guidance for high-risk tasks. In phase 3, the app exceeded success criteria for recruitment, retention, and satisfaction, with participants highlighting its usefulness, usability, and adoption. Qualitative feedback emphasized the app’s practical value as a real-time resource, particularly its step-by-step guidance and user-friendly design.

**Conclusions:**

This study met its objectives, highlighting the SPH MSIP app’s potential to address community health care workers’ unmet safe patient handling needs and improve support in real time patient handling scenarios. While the findings suggest strong feasibility and acceptability, future research should focus on large-scale, extended effectiveness trials to evaluate the app’s impact on reducing musculoskeletal injury rates and improving patient care outcomes.

## Introduction

### Background

Patient handling in health care involves lifting, transferring, repositioning, or mobilizing a patient’s body [1]. These actions—particularly the forward flexion and twisting that often accompany them—place substantial compressive and shear loads on the lumbar and cervical spine [2,3]. Even under ideal conditions, the weight of an adult often exceeds health care professionals’ lifting capacity [2]. Consequently, repeated exposure to high-load and awkward-posture tasks leads to acute sprains and strains of the low back, neck, and shoulders—the most common musculoskeletal injuries linked to patient-handling overexertion [4]. In 2020, the US Bureau of Labor Statistics reported approximately 10,510 workplace injuries in health care due to overexertion and bodily reactions [5]. At the Vancouver Island Health Authority (Island Health) in British Columbia, Canada, from April 1, 2021, to March 31, 2022, patient handling injuries accounted for 25% of all of Island Health’s WorkSafeBC (employer work incident insurance) claims, at a cost of approximately US $3.0 million and a total of 21,286 worker time-loss days. The majority of these claims involved musculoskeletal sprains and strains resulting from unsafe patient-handling maneuvers, confirming that manual handling remains one of the leading injury mechanisms among Island Health’s direct-care workforce.

Community health care workers provide personal care and daily-living support that routinely involves patient handling, often in clients’ homes where access to safety resources and handling tools is limited [6]. They face multiple unmet needs related to safe patient handling, including concerns about the lack of refresher training and a general “get it done” attitude among co-workers that may discourage safe practices [7,8]. This lack of accessible safety resources and support, combined with the challenging environments in which the community health care workers work, further increases their vulnerability to injury. Addressing these gaps is essential to prevent musculoskeletal injuries among community health care workers and to reduce medical costs and the broader burden on health care systems.

A growing body of research shows that interventions can promote safe patient handling and reduce musculoskeletal injuries among health care workers [9], including community health care workers [10]. A systematic review by Wåhlin et al [9] identified 4 categories of successful interventions: the provision and training of assistive equipment (eg, mechanical body lifts), peer coaching programs, physical exercise initiatives, and cultivating a workplace culture with strong management support. Interventions that provide continuous support, rather than one-time training, are more effective [11]. Successful programs not only reduce injuries but also yield cost savings, with studies reporting reduced claims and recurrences after implementation [11-13]. Additionally, emerging technologies, such as real-time audio biofeedback [14] and vibrotactile feedback on patient handling [15], show promise for further enhancing safety practices. However, the practicality and cost-effectiveness of these technologies in real-world health care settings remain uncertain, and their development has not consistently involved feedback from health care workers themselves. Given these proven benefits, despite the constraints of in-person interventions, there is a strong rationale for developing an accessible and effective safe patient handling program tailored to support community health care workers. A digital health intervention, such as a mobile health (mHealth) app, could deliver real-time training, resources, and tools—overcoming logistical barriers and providing continuous support in diverse and often isolated work environments [16,17].

Existing apps such as eUlift [18], Safe Patient Handling [19], and Pain Point [20] illustrate the potential of digital tools, but do not address the realities of community health care workers who provide care in clients’ homes, nor do they align with jurisdiction-specific musculoskeletal injury prevention policies; most also lack user-centered co-design with community health care workers and have not been evaluated in this population [10]. Despite extensive guidance and training programs, few tools are designed for community health care workers operating in clients’ homes, where equipment is limited, workflows are mobile, refresher training is irregular, and peer norms can discourage best practice [7,8]. This creates a practical need for a low-burden, user-centered microcoaching application that provides context-specific guidance and resources within health-system constraints. This study addresses this gap.

### This Study

Evidence-based interventions have demonstrated their efficacy in promoting safe patient handling [9,13], yet logistical and resource barriers often limit their accessibility, particularly for community health care workers in isolated settings [6]. Island Health, Canada, has established the musculoskeletal injury prevention team to promote safe patient handling practices, but its capacity is constrained by the team’s small size, the large number of care workers, and the geographical spread of service areas. Despite successfully implementing guidelines, tools, and in-person training, these efforts are limited by costs and logistical challenges [21].

This study aimed to (1) provide detailed insights into the unmet needs of Island Health community health care workers with respect to safe patient handling resources and access to information, (2) translate those needs into a user-centered prototype of the Safe Patient Handling Musculoskeletal Injury-Prevention (SPH MSIP) smartphone app through an iterative co-design process, and (3) establish the acceptability and feasibility of the SPH MSIP app to support community health care workers’ safe patient handling practices using a mixed methods design. To our knowledge, this is the first app to offer on-demand safe patient handling tools specifically for community health care workers in their work environments. The study also lays the groundwork for testing the hypothesis that on-demand digital coaching, through videos and support tools, can reduce the frequency and severity of musculoskeletal injuries, as well as related WorkSafeBC claims and costs.

## Methods

### Methodological Orientation and Operationalization

We adopted a pragmatist orientation to prioritize answers that are actionable for decision-making in this health-system context [22]. Accordingly, we used a mixed methods single-group feasibility design with prespecified progression criteria to judge whether a larger trial is warranted, and we paired brief quantitative signals with qualitative explanations to understand why those signals emerged [23,24].

In addition to pragmatism, this research adopted a Patient-Oriented Research approach [24]. Patient-oriented research spans information-sharing, consultation/collaboration, shared decision-making/full partnership, and patient-led models [24,25]. Since it increases relevance and acceptability, it enhances uptake and dissemination [24,26]. Aligned with this approach, we engaged musculoskeletal injury prevention coaches, app developers, health system decision-makers, and community health care workers with lived experience of musculoskeletal (patient-handling) injuries. This multidisciplinary team optimized the design and feasibility of the SPH MSIP app and supported co-design, implementation, and adoption.

The research methods were further guided by the IDEAS framework [27], a user-centered design process for developing and evaluating digital interventions. IDEAS is an acronym for its 4 overarching stages: Integrate, Design, Assess, and Share, encompassing 10 phases: empathize, specify, ground, ideate, prototype, gather, build, pilot, evaluate, and share [27]. This study specifically addressed the first 3 overarching stages of the IDEAS framework, encompassing several of the framework’s phases: Integrate (ie, empathize, specify, and ground), Design (ie, ideate, prototype, gather, and build), and Assess (ie, pilot). It also emphasizes the integration of behavioral theory. The Technology Acceptance Model (TAM; [28-30] was selected to inform the development of research questions and guide data interpretation. The TAM [28] posits that perceived usefulness (belief that a technology enhances performance) and perceived ease of use (belief that it is effortless to use) influence attitudes, behavioral intentions, and adoption [29,30]. These constructs provide a practical framework for designing and evaluating user-centered tools [31], and are particularly relevant for assessing the SPH MSIP app. Widely applied in mHealth and app development [32,33], TAM has proven effective in identifying key factors that drive technology acceptance and sustained use in health care settings.

### Study Phase Design

Consistent with our pragmatist and Patient-Oriented Research approach, and using IDEAS to structure activities, we organized 3 phases: phase 1 needs-finding with end users, phase 2 iterative co-design of the SPH MSIP prototype, and phase 3 a single-group mixed methods feasibility study with predefined progression criteria. Specifically, phase 1 merged theoretical insights with end user input to identify unmet safe-patient-handling needs among Island Health community health care workers (objective 1). Phase 2 used iterative co-design with the patient-oriented research team and the app developer to transform those needs into a functional SPH MSIP prototype (objective 2). Phase 3 assessed the prototype’s feasibility and acceptability (objective 3) in a single-group mixed methods study [34], combining postintervention questionnaires with qualitative interviews and survey items to contextualize the numbers. While quantitative methods were the primary focus, qualitative data enriched the understanding of participants’ experiences. To evaluate feasibility, the study established predefined success criteria, including an enrollment rate of ≥50% and a retention rate of ≥75%. Acceptability was measured using a participant satisfaction threshold of a mean score ≥4 points on a 7-point Likert scale.

### Ethical Considerations

Ethical approval was obtained from the University of Victoria Human Research Ethics Board and harmonized ethics approval by Research Ethics BC (REBC#: H23-00322). All participants were adults and provided written, electronic informed consent before any procedures; participation was voluntary, and participants could withdraw at any time or skip any question without penalty. All questionnaire and interview responses were collected in a secure web-based survey platform and Zoom (Zoom Communications), respectively, immediately exported, and stored on a password-protected server in the Behavioural Medicine Laboratory at the University of Victoria. A unique study ID (not names or e-mail addresses) was assigned at first contact; the reidentification key is kept in a separate, encrypted file accessible only to the principal investigator and project coordinator. Audio files were deleted after verbatim transcription. Deidentified analytic files will be retained for 5 years, after which they will be permanently erased from the secure server. The use of the SPH MSIP app during client care was not required; participants were directed to follow employer phone-use and privacy policies and to access content outside client interactions where possible. The app contains training content only and does not collect audio, images, GPS, or client-identifying information. The app was installed on employer-issued work phones.

### Phase 1: Patient-Oriented Research Team Building, Unmet Needs Insight, and Engagement

#### Eligibility Criteria of Participants

The first expressions of interest outlined the patient-oriented research team roles and responsibilities, and 6 employees were selected to participate as members. They consisted of 3 musculoskeletal injury prevention team coaches and 3 community health care workers. They were selected as per the following eligibility criteria. Musculoskeletal injury prevention team coaches qualified based on the following: (1) aged 19 years and older, (2) have a minimum of 12 months of active direct care employment with a Community Health Services (CHS) program at Island Health, (3) have experienced a workplace musculoskeletal injury, (4) are comfortable speaking and reading English, (5) have an Island Health smart phone, and (6) have been a musculoskeletal injury prevention team coach for a minimum of 12 months. The community health care workers qualified based on the following: (1) aged 19 years and older, (2) have a minimum of 12 months of active direct care employment with a CHS program at Island Health, (3) have experienced a workplace musculoskeletal injury, (4) are comfortable speaking and reading English, (5) have an Island Health smart phone, and (6) have used an musculoskeletal injury prevention team coach in the past 12 months. No additional exclusion criteria were applied for these 2 groups of participants. The research team consisted of one professor with research experience in health psychology, feasibility research designs, and a project coordinator.

#### Procedure

The research team hosted 3 digital meetings (totaling 5.5 hours) with the patient-oriented research team. The purpose of meeting 1 was team building, providing an overview of the existing Island Health safe patient handling tools, and gathering initial feedback to better understand unmet needs related to safe patient handling resources and information access. This meeting encouraged engagement from all team members and allowed them to share insights and experiences. In meeting 2, the patient-oriented research team reviewed and selected specific safe patient handling tools for inclusion in the SPH MSIP app, discussing each tool’s relevance and usability, while also providing suggestions for additional design and functionality features to ensure the app would effectively address identified needs. Based on the TAM [28], the research team focused on collecting feedback related to perceived usefulness (eg, how selected tools could enhance safety practices and reduce injury risks) and perceived ease of use (eg, identifying user-friendly formats for app features). This ensured that user insights guided design decisions, promoting greater acceptance of the app. Meeting 3 was dedicated to finalizing both the selection of safe patient handling tools and the unmet needs assessment. TAM [28] was again applied to prioritize features that enhanced user satisfaction, aligned with community health care workers’ workflow, and addressed barriers to adoption, such as usability and resource accessibility. All meeting discussions were recorded, transcribed, and summarized in a report, which was submitted to Pathverse [35,36], the company responsible for developing the SPH MSIP app. This phase established a clear, user-informed foundation for the app’s design, aligned with the priorities and insights of the patient-oriented research team.

### Phase 2: App Development

In phase 2, a series of 4 meetings was conducted, during which the patient-oriented research team collaborated with Pathverse to coideate and codevelop the SPH MSIP app prototype. The team reviewed the selected safe patient handling tools in detail with the app developer, allowing for targeted discussions about integrating these tools into the app and addressing technical considerations. Feedback from this session guided initial design choices. Then, Pathverse incorporated patient-oriented research team feedback into the evolving prototype, presenting updated versions for further review and input. Using TAM [28,30] as a guiding framework, these successive cycles emphasized the enhancement of perceived usefulness and ease of use, while iterative design revisions focused on ensuring the app’s compatibility with users’ work environments. The collaborative approach prioritized the alignment of app features with user needs, fostering positive attitudes toward adoption and usage intentions. By the final meeting, the prototype had been refined and finalized, fully integrating the patient-oriented research team’s insights and meeting the established goals for functionality and user experience. This collaborative approach ensured the app was optimized effectively, resulting in a practical, user-centered tool for safe patient handling support.

### Phase 3: Feasibility Trial

#### Participants

##### Eligibility Criteria

Invitations were sent out to employees working in Island Health. The community health care workers qualified for participation based on the following eligibility criteria: (1) aged 19 years and older; (2) a minimum of 12 months of active direct care employment with a CHS program at Island Health; (3) preferably, have experienced a workplace musculoskeletal injury; (4) comfortable speaking and reading English; (5) own an Island Health smartphone; and (6) preferably, have used an musculoskeletal injury prevention team coach within the past 12 months. Community health care workers’ musculoskeletal injury prevention team coaches were required to meet the same criteria, with the additional qualification of having served as a musculoskeletal injury prevention team coach for at least 12 months. No additional exclusion criteria were applied.

##### Procedure

The study took place from September to October 2023. Upon receiving consent, participants attended a 1-hour orientation and training session in September 2023, where they were introduced to the SPH MSIP app and prepared for the study. Participation was entirely voluntary, and participants were informed of their right to withdraw from the study at any time without consequence. If they chose to withdraw before completing the one-month study period, they could decide whether their data collected up to that point would be included. Participants were asked to use the SPH MSIP app for one month, from October 1 to October 31, 2023, on their work smartphones. The app provided them with on-demand, real-time videos and resources as a resource for safe patient handling in their work environments. At the end of the month, participants completed a follow-up questionnaire and participated in one of 4 small group interviews to discuss their experiences with the app. In recognition of their time, participants received in-kind wage compensation through their regular CHS programs, with additional reimbursement for travel and mileage expenses as needed. Each participant who completed the study also received an honorarium as a token of appreciation.

##### Intervention Materials

The SPH MSIP app was developed in response to the unmet needs identified in phase 1, serving as a comprehensive and user-friendly tool to support community health care workers in implementing safe patient handling practices. Guided by the TAM [28,30], the app incorporated 3 primary intervention components designed to address mechanisms of action related to perceived usefulness, perceived ease of use, and behavioral intention, ensuring the tool was practical, engaging, and compatible with the workflows of community health care workers.

#### Just-in-Time Training Videos

The app provided short instructional videos, each no longer than 1 minute and 30 seconds, offering real-time guidance for safe patient handling tasks. These videos emphasized key practices, such as completing a “Point of Care Patient Handling Risk Assessment” and demonstrating safe techniques like boosting in bed or using patient handling equipment. The videos were designed to enhance perceived usefulness by providing actionable, context-specific guidance, while also addressing perceived ease of use through intuitive accessibility. Videos were accessible through scrolling the main page or via a tailored algorithm, which guided users through a 3-question decision tree to recommend the most relevant video for their scenario. Additional tips, such as reminders to adopt the “STABLE” posture, were provided before task execution.

#### Standard Procedure and Safe Practice Documents

A dedicated resource section housed mobile-friendly PDF documents, including key guides like the “Pre-Standing Safety Check” and information on “Musculoskeletal Injuries Risk Factors.” These materials provided written reinforcement of safe patient handling protocols, enabling community health care workers to review and confirm best practices in a clear, accessible format. This feature was designed to improve the perceived usefulness of the SPH MSIP app [30].

#### Frequently Asked Questions

The app included a robust frequently asked questions section developed collaboratively with the patient-oriented research team in phases 1 and 2. This feature addressed common questions on safe patient handling practices and processes, such as when to use a transfer belt or how to manage ergonomic hazards during client care tasks like donning compression stockings. This component enhanced perceived ease of use by proactively resolving potential barriers to app engagement and reinforced behavioral intention by ensuring the app aligned with users’ practical concerns.

By integrating these TAM-guided features [28], the SPH MSIP app was designed to function as an all-in-one pocket resource, providing perceived usefulness through its comprehensive content, perceived ease of use via user-friendly navigation and personalization, and fostering behavioral intention to adopt and sustain app use. Detailed intervention materials are summarized in Table 1.

**Table 1:** Safe Patient Handling Musculoskeletal Injury-Prevention (SPH MSIP) app contents.

Intervention components	Examples
Just-in-time training videos (25 modules)	One Person Transfer One Person Slide and Turn Away One Person Turn Towards Two Person Head of the Bed Boost Two Person Side of the Bed Boost Two Person Kneeling on Bed Boost Wheelchair Reposition: One Person Seated Scoot Wheelchair Reposition: Two Person Raise and Roll Wheelchair Reposition: Two Person Drawsheet Method Using a Repositioning Sling Using a Universal Sling Using the Band Sling Using a Hammock Sling Strap Configurations Using the SARA Stedy Using the SARA 3000
Standard procedure and safe practice documents (6 PDF resources)	Bed Reposition: Slide to the side STABLE: Tips to help reduce injuries Point of Care Risk Assessment Pre-Standing Safety Check
Frequently asked questions (33 questions and answers)	Q: What is the recommended weight limit for manually lifting clients or parts of clients in ideal conditions? A: 16 kgs (35lbs). The goal of safe client handling programs should be to eliminate all manual lifting whenever possible. Q: Should a bed be height adjustable? A: A client may need to be assessed by a Community Health Services clinician for a height adjustable bed to ensure workers can achieve safe working heights for all required tasks Q: What is general safe working height for heavy work? A: Heavy work requires large amounts of force. Generally a working height of 65-95 cm (23-37.5 inches) is considered safe for heavy work. Examples of heavy work could include: boosting, turning or repositioning clients. Q: When do I use a transfer/gait belt? A: Must be used during every manually assisted transfer of a client. Examples of these situations include but are not limited to: standby assist, one- or two-person transfer, wheelchair to chair, chair to toilet or commode, bed to wheelchair with or without a transfer pole, walking with or without a walking aid, when a patient is at risk of falls and repositioning in a wheelchair. Q: How do I secure the transfer/fait belt on a client? A: The belt should be fastened securely around the hips of the client. It should be snug but still comfortable. You should be able to place one or two finger widths snugly between the client and the belt when tight. Q: When do I NOT use a transfer/gait belt? A: A transfer/gait belt should never be used to “lift” a client. And these belts are NOT to be worn by care providers as a handhold for the client. Q: What general ergonomic hazards when I assist clients to don medically prescribed compression stockings? A: Donning of compression stockings is a hazard for workers due to the ergonomic risk factors involving: awkward postures, forces and static postures. Q: What is equipment best practice for assisting clients to don medically prescribed compression stockings? A: Clients who require assistance with donning and doffing of compression stockings when the compression is greater than 20mmHg should have appropriate donning and doffing equipment available and accessible for CHW use. Q: What’s considered safe floor transitions? A: Transitions between floor types are smooth with minimal gaps or changes in height. Q: What are the recommendations for wheels and castors? A: The type of wheels and castors attached to wheeled equipment must be appropriate for the type of flooring present to reduce ergonomic risk.

### Measures

#### Quantitative Outcomes

##### Recruitment and Retention

*Recruitment* rate was calculated by dividing the number of participants who expressed interest in the study by the total number of spots available for the study. Retention was calculated by dividing the number of participants who completed the final questionnaire and exit interview by the number of participants who attended the prestudy orientation sessions.

##### Satisfaction, Acceptability, and Feasibility

A SPH MSIP app-specific TAM questionnaire was developed and adapted based on existing studies using TAM [28,30,37] to evaluate the participant experiences with the SPH MSIP app after the 1-month trial period. Satisfaction and feasibility were measured using a 7-item satisfaction and evaluation questionnaire on a 7-point Likert scale, ranging from 1 (Strongly Disagree) to 7 (Strongly Agree). Sample items were “Overall I enjoyed using the Safe Patient Handling MSIP Application,” “If the SPH MSIP APP was available after this I would continue to use it,” “I am open to using technology like the SPH Virtual Coach APP,” and “It makes sense for me to use the SPH Virtual Coach APP in my work life.”

#### Qualitative Outcomes

**Figure 1:**
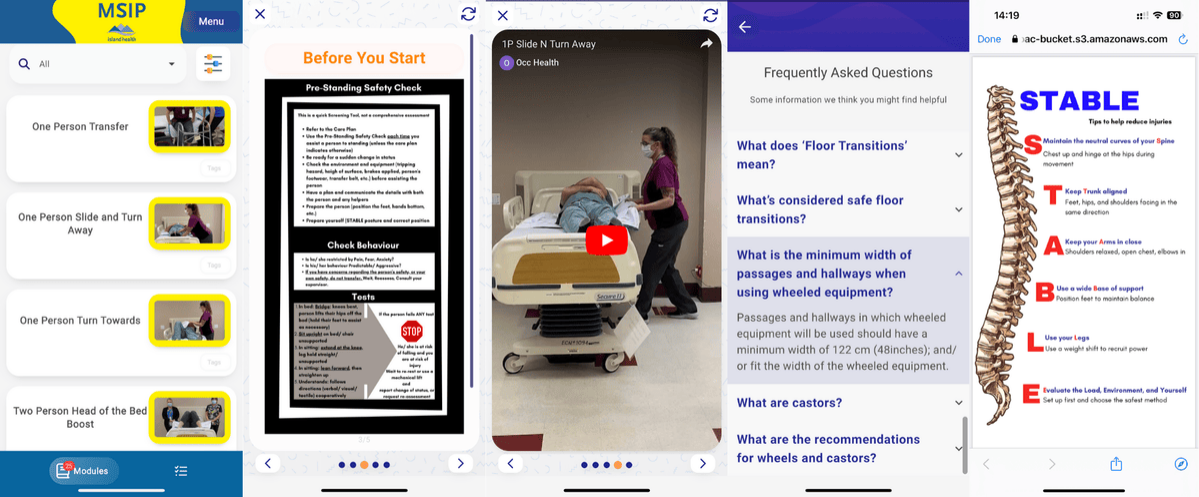
Screenshots of Safe Patient Handling Musculoskeletal Injury-Prevention (SPH MSIP) app.

Qualitative data retrieval was completed using a small group interview-based approach (4 sessions) with participants over a 2-week period (November 2023). This data was used to further understand the quantitative outcomes. The interview was conducted using the digital conferencing platform Zoom and lasted approximately one hour. Interviews were conducted by 2 different interviewers. Both interviewers were trained and engaged in practice interviews prior to interviewing participants. Interviews were transcribed verbatim by multiple research assistants. These sessions explored TAM constructs [28,37], including (1) perceived usefulness/appropriateness/demand (eg, “Do you think this app is useful in reducing workplace MS Injury risk for yourself?”), (2) perceived ease of use (eg, “Are there certain features that made the app more usable? If yes, what are they?”), (3) perceived enjoyment/satisfaction (eg, “What, if anything, could contribute to making the app more enjoyable for you to use?”), (4) Intention to use/continue use (eg, “Can you see yourself using the app beyond this study?”), (5) actual use (eg, “How often did you use/look at the app in a week? Or in the month?”), (6) compatibility (eg, “Does using the app fit into your current work lifestyle?”), (7) attitude (eg, “Is there any apprehension you feel about using this app while working with clients?”), and (8) self-efficacy (eg, “How confident do you feel using an app like this in your workplace daily?”). The complete interview guide was included as Figure 1.

### Data Analysis

#### Quantitative Outcomes

The rating system established for quantitative data analysis was based on the traffic light system from Lewis et al [38], which recommends that progression be based on all key feasibility criteria, with the decision ultimately being based on the worst-performing criterion. The AMBER (middle) zone may be divided into 2 cutoff points to indicate when minor or major amendments are necessary prior to proceeding [38].

#### Qualitative Outcomes

Interviews were audio-recorded, transcribed verbatim, and analyzed in Excel by AL and SW to identify common themes regarding the app’s strengths and limitations. Using a constructivist approach [39], this analysis acknowledged that knowledge is coconstructed between researcher and participant, with meaning derived through interpretation rather than objective discovery. Following Braun and Clarke’s [40] reflexive thematic analysis, AL and SW began by familiarizing themselves with the transcripts and open-ended responses, reading closely to capture initial impressions. They conducted initial coding to categorize data based on broad aspects such as feasibility, acceptability, and intervention outcomes, grouping these codes into preliminary themes and subthemes. These preliminary groupings were then presented to SB, SC, and RR (supervisor) for feedback to refine the thematic structure. Following discussions, the team refined and finalized the themes, which captured participants’ views on the app. Representative quotes were then selected to highlight these themes, providing concrete examples of the app’s perceived strengths and limitations. This iterative process enabled a comprehensive understanding of participants’ experiences, supported by both individual coding and collaborative reflection.

## Results

### Results of Phase 1

Based on phase 1 meetings, we identified key unmet needs among community health care workers related to safe patient handling and injury prevention, as well as specific feedback on safe patient handling tools that could enhance the SPH MSIP APP under development. Several major concerns and needs were expressed (Textbox 1 for details), including the high risk of injury due to client care tasks, frequent work in isolation at client homes, and a tendency to prioritize client needs over personal safety. Furthermore, gaps in safe patient handling knowledge and practices were noted, such as limited access to training on point-of-care risk assessment and challenges in obtaining refresher training on safe handling practices. Additionally, a “get it done” culture in the workplace, alongside inconsistent proficiency among leaders in safe handling techniques, emerged as a significant issue affecting community health care workers’ safety and effectiveness. Notably, participants also highlighted specific high-risk patient handling activities that require additional support and clear guidance, including boosting clients in bed, assisting clients with compression stockings, providing care in low or bariatric beds, and assisting clients with walking in confined spaces. Essential equipment used in these tasks, such as slings, transfer belts, and slider sheets, requires proper usage training, and community health care workers expressed a need for accessible, step-by-step instructions. In addition, it was made clear by participants that the app needed to address practical questions that community health care workers often face, such as “How do I best use my body in this situation?” and “What is the best practice for this specific patient care task?” Overall, community health care workers emphasized that the app should provide easily accessible, concise, and practical information that could be accessed without disrupting client interactions.

**Phase 1 feedback from community health care workers.**

**Unmet needs identified by community health care workers in phase 1**

- Concerns of general potential for client care and handling injury
- Concerns of injuries can happen fast
- Concerns of often working alone in clients’ homes to provide care
- Gaps in knowledge regarding safe practice, safe assist weight, safe work expectations
- Gaps in knowledge and use of point-of-care risk assessments
- Concerns that co-workers become injured
- Concerns regarding “get it done” attitude
- Concerns of “problem solve on the spot; alone”
- Concerns of “even the nurse leaders aren’t always proficient on safe patient handling and techniques”
- Community health care workers—very generous meeting client needs and sometimes push themselves beyond what is physically safe—putting the client first
- Concerns of community health care workers are in client’s homes—a more intimate and personal environment
- Concerns of creating appropriate work boundaries in client’s homes
- Concerns about no safe patient handling refresher training easily available for employees
- When have in person refresher training it is time consuming
- Access to the prehandling safety check tool in the moment
- Communication routes for reminders, refreshers, updated safe practice information that is easy to access and review quickly
- Top high risk patient handling activities:
  - Bed boosts
  - TED stockings/compression stockings
  - Bariatric bed care
  - Assisted walking clients with transfer belt
  - Showering clients in tight spaces
  - Low beds
  - Standing peri-care
- Top equipment used:
  - Slings
  - Slider sheets
  - Sara Stedy
  - Overhead lift systems
  - Floor lift systems
  - Hospital beds
  - Etac Turner (standing pivot disc)
  - EZ disc
  - Transfer belts
  - Transfer board
  - Bed Ladders (rare)
  - Sask-a-pole (transfer pole)
- Work Smartphones: I would just like our phones to actually work- better connectivity
- Worker can be some lack of comfort pulling out and using phone in front of the client (clients might be suspicious/ added confusion/ do not understand)
- Practice questions (concerns):
  - How do I use this piece of equipment? Example: sara stedy
  - How do I best use my body in this situation?
  - What is best practice for this pt care and handlings situation?
- Training resources for teaching/co-working with client family members
- App must be easy to navigate
- Concerns about “language barriers” for employees/clients using the App. Some employees have hard time with English
- Need clear answers/ or clear video or clear explanation on the App that does not need to be reinterpreted or reclarified
- Easy to access information and resources and to maneuver through the information in a timely way. Simple and fast

### Results of Phase 2

Building on the unmet needs identified in phase 1, phase 2 focused on designing a user-centered SPH MSIP APP tailored to community health care workers’ challenges in safe patient handling practices (Table 2). Collaborating with Pathverse, the app addressed critical gaps in safe patient handling knowledge, training accessibility, and the unique challenges of working in isolated client homes. The app provided clear, practical guidelines for high-risk tasks such as boosting clients in bed, assisting with compression stockings, and managing bariatric or low beds, as well as proper use of commonly used equipment like slings, transfer belts, and slider sheets. Tools such as point-of-care risk assessment and pre-standing safety check guides were also included to support safety and decision-making. The SPH MSIP APP improved training accessibility with mobile-friendly resources and concise, scenario-specific instructional videos. A tailored algorithm streamlined access to relevant materials, minimizing disruptions during client care. Although some tools and equipment (eg, Sara Stedy, Etac Turner) fell outside the project’s scope, most high-priority resources were successfully integrated, offering a reliable and practical resource to enhance community health care workers’ safe patient handling practices. Educational content (training videos, PDF, and equipment checklists) was adapted directly from Island Health’s Occupational Health and Safety safe-patient-handling resources. All materials were produced or reviewed by MSIP coaches, occupational health and safety specialists, and professionals to ensure they reflected standard device use and current workplace guidelines before being embedded in the app [41,42]. Screenshots of the SPH MSIP APP can be found in Figure 1.

**Table 2:** Phase 2 unmet needs addressed.

Unmet needs identified in phase 1 by the patient-oriented research team	How we addressed unmet needs in phase 2
Gaps in knowledge regarding safe practice, safe assist weight, safe work expectations	Provided SPH MSIP^a^ app
Gaps in knowledge and use of point-of-care risk assessments	Included point-of-care risk assessments in SPH MSIP app
Concerns of ‘problem solve on the spot; alone”	Provided SPH MSIP app
Concerns of “even the nurse leaders aren’t always proficient on safe patient handling and techniques”	Provided SPH MSIP app and invitation to share experience with other colleagues and leaders
Concerns: Community health care workers are in client’s homes—a more intimate and personal environment	Provided SPH MSIP app
Concerns of creating appropriate work boundaries in client’s homes	Provided point-of-care risk assessments in SPH MSIP app
Concerns about no safe patient handling refresher training easily available for employees	Provided SPH MSIP app
When have in person refresher training it is time consuming	Provided SPH MSIP app
Access to the pre-handling safety check tool in the moment	Included Pre-handling Safety Check tool in SPH MSIP app
Communication routes for reminders, refreshers, updated safe practice information that is easy to access and review quickly	Provided SPH MSIP app
Top high risk patient handling activities: Bed boosts. TED stockings/compression stockings Bariatric bed care Assisted walking clients with transfer belt. Showering clients in tight spaces Low beds Standing peri-care	Included information or guidelines for all activities in SPH MSIP app Except standing peri-care and showering clients in tight spaces
Top equipment used: Slings Slider sheets Sara Stedy Overhead lift systems Floor lift systems Hospital beds Etac Turner (standing pivot disc) EZ disc Transfer belts Transfer board Bed Ladders (rare) Sask-a-pole (transfer pole)	Included information or guidelines for all equipment listed in SPH MSIP app Except Etac Turner, Sara Stedy, EZ Disc, Transfer Board, Transfer pole
Work Smartphones: I would just like our phones to actually work—better connectivity	Ensured Island Health IM/IT and Pathverse provided best service for use of SPH MSIP app
Worker can be some lack of comfort pulling out and using phone in front of the client (clients might be suspicious/ added confusion/ do not understand)	Ensured resources in SPH MSIP app were short duration and easy to view
Practice questions (concerns): How do I use this piece of equipment? (Example: sara stedy). How do I best use my body in this situation? What is best practice for this pt care and handlings situation?	Provided resources in SPH MSIP app for practice questions in the videos, frequently asked questions, resources sections
Training resources for teaching/co-working with client family members	Provided SPH MSIP app and encouraged to share with colleagues for training purposes
App must be easy to navigate	Provided insights to Pathverse, the app development company
Need clear answers/or clear video or clear explanation on the app that does not need to be reinterpreted or reclarified	Provided best in case videos and resources in SPH MSIP app
Easy to access information and resources and to maneuver through the information in timely way. Simple and fast.	Provided SPH MSIP app without barriers to entry to Pathverse app platform once loaded on work smartphone

^a^SPH MSIP: Safe Patient Handling Musculoskeletal Injury-Prevention.

### Results of Phase 3

#### Quantitative Outcomes

##### Recruitment and Retention

Thirty-two eligible staff members were invited, and 28 out of 32 (88%) participants enrolled—14 community health care workers and 14 community health care workers musculoskeletal injury prevention team coaches—from Port Alberni, Cowichan, Nanaimo, and Comox. Of those enrolled, 26 out of 28 (93%) participants completed all aspects of the study, with 2 completing the questionnaires but not the exit interview. The recruitment and retention rates exceeded the predefined success criteria (Table 3).

**Table 3:** Participant and peer mentor intervention feasibility and acceptability success criteria.

Outcome	Success criteria	Results
Enrollment rate, %	≥50	88
Retention rate, %	≥75	93
Satisfaction score (points), mean	≥4	>5.96

##### Satisfaction

Enjoyment of the SPH-MSIP app was high: 22 out of 28 (79%) participants agreed or strongly agreed that they enjoyed using it, 3 out of 28 (11%) participants somewhat agreed, and 3 out of 28 (11%) participants somewhat disagreed. This resulted in a mean score of 5.96 out of 7, significantly exceeding the predefined success criterion of a mean score of 4 (Table 3).

##### Acceptability and Feasibility

Participants expressed a strong overall willingness to use the SPH MSIP APP in the future, should it remain available after the study. A total of 23 (82%) participants agreed or strongly agreed with continuing to use the app. Openness to using similar technology in the future was even higher, with 26 participants (93%) agreeing or strongly agreeing. A majority—24 of 28 (86%) participants—felt that using the app in their future work life made sense, and 20 (71%) participants agreed or strongly agreed that it fit well into their workflow. Confidence in learning to use the app in the future was high, with 25 (89%) participants agreeing or strongly agreeing. Participants’ belief in their ability to use the app was equally strong, with 25 (89%) participants agreeing or strongly agreeing that they had the necessary skills. There were no “strongly disagree” responses and only a small number of “disagree” responses across all items. Notably, one participant (1/28, 4%) disagreed with continuing to use the app if available, and one participant (1/28, 4%) disagreed with confidence about learning to use the app (Table 4).

**Table 4:** SPH MSIP app satisfaction and feasibility.

Questions	Strongly agree, n (%)^a^	Agree, n (%)^a^	Somewhat agree, n (%)^a^	Neither agree nor disagree, n (%)^a^	Somewhat disagree, n (%)^a^	Disagree, n (%)^a^	Strongly disagree, n (%)^a^	Mean (SD)
1. Overall, I enjoyed using the SPH MSIP app	11 (39)	11 (39)	3 (11)	—^b^	3 (11)	—	—	5.96 (1.23)
2. If the SPH MSIP app was available after this, I would continue to use it	15 (54)	8 (29)	3 (11)	—	1 (4)	1(4)	—	6.18 (1.25)
3. I am open to using technology like the SPH Virtual Coach app	15 (54)	11 (39)	1 (4)	—	1 (4)	—	—	6.36 (1.03)
4. It makes sense for me to use the SPH Virtual Coach app in my work life	12 (43)	12 (43)	3 (11)	1 (4)	—	—	—	6.25 (0.80)
5. I feel confident about learning to use the SPH Virtual Coach app	11 (39)	14 (50)	1 (4	1 (4)	—	1 (4)	—	6.14 (1.08)
6. I have the necessary skills to use the SPH Virtual Coach app	12 (43)	13 (46)	2 (8)	—	1 (4)	—	—	6.25 (0.89)
7. Using the SPH MSIP app is compatible with most aspects of my work life	4 (14)	16 (57)	5 (18)	2 (8)	1 (4)	—	—	5.71 (0.94)

^a^Percentages may not total 100% due to rounding.

^b^Indicates 0 responses (0%).

#### Qualitative Outcomes

##### Overview

Qualitative data were collected from 26 participants through small group interviews conducted over 4 sessions in a 2-week period. In general, the participants found the SPH MSIP APP to be an impactful tool in supporting their roles as community health care workers and musculoskeletal injury prevention coaches. The themes, subthemes, and selected quotes are presented in Table 5. Many participants described the app as a “game changer,” emphasizing its ease of use, the relevance of its content, and its ability to enhance their confidence in executing care tasks safely and efficiently. They highlighted its effectiveness in bridging gaps in knowledge and its value as a resource for immediate reference, skill refreshment, and peer coaching. Nonetheless, participants also offered constructive feedback for improvement, including suggestions for enhancing app functionality, content comprehensiveness, and integration into their daily routines. Thematic analysis revealed 3 major themes—usefulness, usability, and adoption—each comprising several subthemes that encapsulate participants’ feedback and reflections on the SPH MSIP app.

**Table 5:** Themes, subthemes, sub-subthemes, and selected quotes supporting the themes.

Main themes, subthemes, and sub-subthemes				Examples of quotations from participants
**Usefulness**				
	**Perceived effectiveness**			
		Pros	It was nice to be able to review prior to coaching someone or when somebody was asking something, I was able to pull it out and show them what I meant; that made a really big difference. [SPA015] It's easier for in the moment to show your peer or colleague that you're working with. As a coach before I'd have to go back to the office and email the how to or video or link. And so it's nice to have it right there. [SPA009] It perhaps might remind you to be a little more present and mindful. These videos really kind of force you to pay more attention. Slow down a bit, be a little more mindful of all the little details that are being pushed in these videos that perhaps you might become complacent to [SPA008]	
		Cons	(How does using the app compare to your experience using a human Musculoskeletal Injury Prevention team coach to support you in your workplace?) It's a great tool to use as support for a Musculoskeletal Injury Prevention team coach coming in, but not as a replacement for a coach. Having a human watching you and seeing where your body mechanics are is very valuable. [SPA025]	
	**Content assessment**			
		Pros	I really appreciate that the videos, were short and sweet. Sometimes we don't have a lot of time to go it was just right to the point [SPA020] The video is really helpful. People tend to be visual. We only have a few minutes of our time just to check on that phone so we are able to imagine how we can do it. So, the video is more helpful for me. [SPA005] The frequently asked questions I found very useful, they're pretty comprehensive [SPA019]	
		Cons	(What type of content was missing from the app that you would like to see included?) Stocking application would've been a good one to have in there, like how to put on TED stockings properly [SPA004] (What type of content was missing from the app that you would like to see included?) Transferring somebody onto a bath bench or a shower bench or a bathing chair. [SPA018] It was weird to have the point of care risk assessment video and then the poster. people might just skip that to get to the point. Maybe it should just be completely together so that it’s repetitive that we do need to do our point of care risk assessment. One of the biggest things is that we could prevent injuries with just that simple tool. I appreciate it's there for every video, but instead of having the two or three little dots that you can slide through, just combine it all into one video. [SPA024]	
**Usability**				
	**Perceived ease of use**			
		Pros	I had one of our Musculoskeletal Injury Prevention team coaches who, she says she's not good with the cell phone. She is my age. It took five minutes with her and that's all she needed [SPA017] I liked how straightforward the app was when I open it up, it is quick and bold and easy to read the different sections [SPA020] It's been a lot easier than navigating than the SharePoint and the intranet pages. So, we used it a lot, when developing plans. So, it's been the ease of access that has been very nice. [SPA009]	
		Cons	Is there a way that the videos can be saved so that we don't have to wait for them? [SPA006] It'd be cool if the videos could be like embedded somewhere that we can access 'em right on our phone without having to access the internet, you couldn't load the videos if you didn't have internet [SPA004] If there was a way to search FAQ'sa, I couldn't find a way to search for a keyword [SPA019]	
	**Compatibility**			
		Pros	We all use our phones for work already, so it's pretty easy to use the app because we do so much work on our phones, it's not that much of a leap to now jump on an app [SPA004] I did show a client a video because of a way that she wanted me to adjust her in the wheelchair and I showed her that we aren't allowed to do it like that, and then I showed her how I was going to do it and she was super receptive to it and then everybody just started doing it like that cause I would show the next worker and the next worker, and then she would tell them this is how we're gonna do it now. [SPA004] I'd like to use it as part of my toolset for coaching. Coaching the CHW's to check this video out quickly before we go in?’ they can prepare themselves for what they're doing. it makes it easier for me to explain where they could improve [SPA025]	
		Cons	(Is there any apprehension you feel about using this app while working with clients?) I did explain to them why as well. So, they're a little bit more open to it after that. But the first reaction was, ‘it's kind of rude being on your phone in my house’ sort of thing. [SPA027] I think the only apprehension I would have is probably time constraint, unfortunately, our time has become shorter and shorter with each client and the only issue I might have is that there might not be time to stop and look at your phone and see what's going on. [SPA007] Some clients don't appreciate us being on our phones when we're in their homes [SPA004]	
**Adoption**				
	**Actual use and frequency**			
		Pros	(How often did you use/look at the app in a week?) I was about twice a week [SPA001] We were using it daily. We're showing everybody we possibly could. We'd bring people down to show them. [SPA002] I was doing quite a bit of coaching in the field around the time we got the app, and it was really helpful because there was no place to demonstrate anything to the CHWs before they came into the client's home. It was a really good way to show them the videos and make sure we are all on the same page in the driveway. Every time I coached, I would show the video. It was every day that I coached. [SPA019]	
	**Intent to use**			
		Pros	I would just like to say that you know, just continuing to use it as an education tool, especially with our new hires. I don't always have a room with a lift and can't go over every piece of part, like we, I can talk about all the slings, I can talk about how they're used, but to be able to have the video and have them pull it up in their own hands and talk about it in the motion [SPA003] (Can you see yourself using the app beyond this study?) Yes - It only makes sense. It's literally like an app to make my job easier. [SPA018] (Can you see yourself using the app beyond this study?) Yes - I think it was great for home support because it's a good refresher. we can go weeks when it's just, basic care, and then all of a sudden, we have a client who has an in-home overhead lift, full bed slider.It's good to look refresh on the app and just see, ‘okay, this is how it's done,’ [SPA001]	
	**Preparedness and confidence**			
		Pros	(What would help you feel more prepared to use the app in the workplace?) Advertise the app around the office [SPA011] (What would help you feel more prepared to use the app in the workplace?) Each department sending out a look to the future kind of email. Like this will coming soon to our Island Health work phones. [SPA018] (How confident do you feel using an app like this in your workplace daily? Scale of 1-5 (5 = extremely confident 1 = not confident at all) Five for confident over here. [SPA007, SPA022, SPA004, and SPA028]	

^a^FAQ: frequently asked questions.

##### Usefulness

The theme of usefulness encompassed participants’ perceptions of the app’s effectiveness and the quality of its content, categorized into 2 subthemes: perceived effectiveness and content assessment.

#### Perceived Effectiveness

Participants described the SPH MSIP APP as “useful,” “very useful,” and “helpful,” with many affirming that it “made a really big difference” in their work. The app was praised as a reliable, consistent, and readily accessible tool for reviewing proper techniques before executing tasks or coaching peers. One participant noted, “These videos really kind of force you to pay more attention, slow down a bit, and be a little more mindful of all the little details that are being emphasized.” The app’s role as a training tool for new hires and employees in CHS was particularly valued, especially by musculoskeletal injury prevention coaches. One coach shared, “It’s easier for in the moment to show your peer or colleague that you’re working with. As a coach before I’d have to go back to the office and email the how to or video or link. And so it’s nice to have it right there.” Despite these advantages, some participants emphasized that the app could not fully replace the role of a human musculoskeletal injury prevention team coach. They highlighted the unique value of having an experienced coach provide personalized feedback and guidance. One participant remarked, “It’s a great tool to use as support for a Musculoskeletal Injury Prevention team coach coming in, but not as a replacement for a coach. Having a human watching you and seeing where your body mechanics are is very valuable.”

#### Content Assessment

The app’s videos received widespread appreciation, with participants praising their clarity and relevance. One participant explained, “The video is really helpful. People tend to be visual. We only have a few minutes of our time just to check on that phone so we are able to imagine how we can do it.” Several participants also acknowledged the usefulness of the frequently asked questions; one participant commented, “The frequently asked questions I found very useful, they’re pretty comprehensive.” However, participants identified areas for improvement, including the addition of videos on tasks such as applying TED stockings or transferring patients to shower benches, “Stocking application would’ve been a good one to have in there, like how to put on TED stockings properly” and “Transferring somebody onto a bath bench or a shower bench or a bathing chair.”

#### Usability

The theme of usability highlighted participants’ experiences with navigating and integrating the app into their workflows, focusing on 2 subthemes: perceived ease of use and compatibility.

#### Perceived Ease of Use

Most participants found the app intuitive and easy to navigate, with one musculoskeletal injury prevention team coach remarking, “I had one of our coaches who, she says she’s not good with the cell phone. She is my age. It took five minutes with her and that’s all she needed.” Compared with existing resources like the Island Health intranet, the app was described as more accessible and user-friendly: “It’s been a lot easier than navigating the SharePoint and the intranet pages. So, we used it a lot, when developing plans. So, it’s been the ease of access that has been very nice.” However, technical issues such as slow video loading and the need for an internet connection to access content were noted: “It’d be cool if the videos could be like embedded somewhere that we can access ‘em right on our phone without having to access the internet, you couldn’t load the videos if you didn't have internet.”

#### Compatibility

The app was praised for its seamless integration into participants’ existing work routines. Community health care workers noted that having the app on their work smartphones aligned well with their frequent use of these devices. As one participant observed, “We all use our phones for work already, so it’s pretty easy to use the app because we do so much work on our phones, it’s not that much of a leap to now jump on an app.” However, some participants encountered challenges, such as concerns about appearing unprofessional when using phones in clients’ homes (“I did explain to the client why as well. So, they are a little bit more open to it after that. But the first reaction was, ‘it’s kind of rude being on your phone in my house’ sort of thing”). The majority of community health care worker participants enjoyed using the SPH MSIP APP. There was a high amount of adoption and usage among this group of community health care worker participants, who were engaged throughout and provided multiple suggestions for continued improvement of content and platform/delivery/experience.

### Adoption

The theme of adoption examined the extent to which participants used the app, their intent to continue using it, and their preparedness and confidence.

#### Actual Use and Frequency

Participants reported using the app with varying frequency, ranging from daily use during coaching sessions to twice a week for quick reference. One coach explained, “It (the app) was a really good way to show them the videos and make sure we are all on the same page in the driveway. Every time I coached, I would show the video. It was every day that I coached.”

#### Intent to Use

Participants expressed strong intent to continue using the app, describing it as a valuable resource for self-reflection, skill refreshment, and task preparation: “Yes - I think it was great for home support because it’s a good refresher.” When asked if they envisioned using the app beyond the study, responses were positive: “Yes - It only makes sense. It’s literally like an app to make my job easier,” accompanied by visual affirmations like thumbs up during group interviews.

#### Preparedness and Confidence

When asked what would help you feel more prepared to use the app in the workplace, some participants said it was already easy enough to use, and some recommended “Advertise the app around the office.” Most participants felt confident using the app, with many rating their confidence as “5” on a scale of 1 to 5. They appreciated the app’s role in instilling confidence not only in their own care practices but also in communicating these practices to clients and their families.

## Discussion

### Overview

This 3-phase formative study showed that a co-designed SPH MSIP smartphone app is both feasible and acceptable for community health care workers. We achieved high recruitment (28/32, 88%), excellent retention (26/28, 93%), and strong user satisfaction/usability (mean 5.96/7), while qualitative feedback highlighted the app’s practicality, clarity, and seamless fit within everyday workflows. This study represents one of the few comprehensive efforts to develop and evaluate an app tailored specifically to the needs of community health care workers.

### Principal Findings

#### Unmet Needs and Community Health Care Workers’ Feedback

Our study revealed multiple unmet needs in safe patient handling resources for community health care workers, highlighting systemic barriers to safe handling practices. Key issues included the lack of accessible refresher training, inconsistent implementation of risk assessments, and workplace cultures that often prioritize efficiency over safety [43]. These findings align with previous research [44], which documented similar challenges in health care environments, where frontline workers face considerable strain due to inadequate support and resources [45]. Notably, the “get it done” attitude observed among coworkers mirrors findings from studies [8,46] emphasizing the disconnect between institutional safety policies and actual practices in health care settings.

This study adopted the Patient-Oriented Research approach, which was instrumental in addressing these barriers through a structured co-design process [24-26]. In this study, the Patient-Oriented Research team actively collaborated with researchers and app developers to shape the SPH MSIP APP, contributing to decisions on content, functionality, and design. This inclusive approach ensured that the app was both practical and relevant, addressing the realities of the workers’ diverse and often challenging environments [25]. The co-design process enabled the research team to incorporate feedback on essential features, such as scenario-specific guidance for high-risk tasks (eg, transferring patients) and intuitive navigation, directly addressing worker needs and enhancing the app’s usability [47]. It also reflects a growing recognition in the literature that digital tools can bridge gaps in on-the-job training by offering immediate, context-relevant support [48,49]. From a TAM perspective [28], the co-design approach prioritized perceived usefulness and ease of use, both critical factors for adoption. For instance, the inclusion of step-by-step instructions for tasks like boosting patients in bed [50] directly addressed the perceived usefulness of the app by boosting workers’ confidence in their handling practices. Likewise, the focus on a user-friendly interface and streamlined workflow integration reflected the importance of ease of use, ensuring that the app could be seamlessly adopted into daily routines.

By integrating the Patient-Oriented Research approach [24-26] and TAM constructs [28], our co-design process showed that engaging end users yields practical, user-centered solutions that are likely to be adopted. This echoes human-factors guidance that rigorous, iterative user-centered design enhances the effectiveness, efficiency, and satisfaction of mHealth technologies [51] and underscores the need for ongoing co-design [47], usability optimization, and feedback loops when scaling to other health regions.

#### Feasibility and Acceptability

The findings from this study suggest that the SPH MSIP APP is a feasible and acceptable intervention for community health care workers and contributes to the growing body of evidence supporting the utility of mHealth tools in health care settings. Consistent with previous studies [52,53], our feasibility results highlight the potential of digital interventions to address specific gaps in training and resource accessibility. Recruitment and retention rates exceeded expectations, and participants provided positive feedback regarding the app’s ease of use, content relevance, and integration into daily workflows. The progression criteria established for this study—encompassing usability, satisfaction, and continued engagement—were consistently met, further underscoring the app’s potential as a practical resource for community health care workers. These results echo findings in the broader literature that highlight the growing acceptance of mHealth interventions among health care providers [54,55], particularly when designed with user-centric principles [56].

The app’s emphasis on real-time accessibility and user-friendly design was particularly appreciated by participants, many of whom noted its potential to enhance confidence in performing safe patient handling tasks. These features align closely with the TAM [28], particularly in terms of perceived ease of use and perceived usefulness. Participants found the app intuitive and valuable, suggesting that it effectively addressed some barriers to safe patient handling practices among community health care workers. However, the short duration of the study limits our understanding of whether these initial impressions would translate into sustained changes in behavior or reductions in musculoskeletal injuries. Previous research [57,58] has shown that the efficacy of digital interventions often diminishes over time without ongoing engagement strategies, suggesting a need for future iterations of the app to incorporate features such as in-app support from peers or coaches, personalized feedback, and periodic updates to maintain user interest [59]. These features would directly enhance the app’s perceived usefulness and ensure that users remain engaged and motivated to incorporate safe handling practices into their routine [28,59]. Moreover, while high satisfaction rates are encouraging, they do not necessarily predict long-term adherence to best practices, highlighting the importance of further research to evaluate the app’s impact on musculoskeletal injury outcomes in more complex, real-world settings. From a TAM perspective [28], while the app’s ease of use and perceived usefulness were apparent, sustained use could depend on enhancing its relevance to users’ evolving needs and providing mechanisms to reinforce behavior change [60,61]. This is where the app could benefit from additional evaluation to refine its features based on longer-term feedback, ensuring it remains useful and engaging beyond initial exposure [62].

While the app shows promise, its development and implementation also underscore the complexities involved in translating research into practice. For instance, the process of enabling the app on community health care workers’ devices revealed structural barriers within Island Health, where restrictive IT policies limited the ease of deployment. This challenge aligns with the concept of perceived ease of use in TAM [28], which suggests that any technical barriers to usability can impede user acceptance. These barriers highlight the importance of considering organizational readiness and infrastructure when designing and scaling mHealth interventions [63]. This finding resonates with recommendations from the World Health Organization [64], which emphasize the need for alignment between technological innovations and institutional support systems to ensure successful implementation. Health authorities looking to implement similar interventions may need to account for potential technological and organizational challenges that can affect the perceived ease of use and, consequently, the overall success of the intervention [63,64].

### Strengths, Limitations, and Future Directions

A key strength of this study is its rigorous, end-to-end co-design process: a multidisciplinary, patient-oriented research team of coaches, frontline workers, developers, and decision-makers guided every phase, ensuring the app matches the realities of community care settings. This collaborative approach translated into exceptional engagement and retention, and every prespecified usability and satisfaction criterion was surpassed. Moreover, the study delivers one of the first comprehensive evaluations of a safe-patient-handling m-health tool purpose-built for community health care workers, addressing a critical gap in the digital-safety literature.

This study also had several limitations that need to be acknowledged and addressed in future research. First, the small, self-selected sample may have introduced selection bias, as those who volunteered may have been more technologically adept or motivated than the broader community health care workers population. To address this, future effectiveness studies should use more inclusive recruitment strategies, potentially incorporating random sampling methods to capture a more representative demographic, and include a control or comparison group (eg, randomized or quasi-experimental design) to enable stronger causal inference [65]. Second, the absence of blinded participatory meetings in phase 1 may have influenced the data collection process, as researchers’ relationships with participants could have biased responses. Future studies should consider using independent facilitators for initial data collection phases to minimize potential biases and ensure the reliability of findings [66]. Furthermore, this feasibility trial did not assess the efficacy of the SPH MSIP app in reducing musculoskeletal injuries. A fully powered, longer-term randomized controlled trial is the logical next step to evaluate whether the app can lower musculoskeletal injury rates, lost-time claims, and related disability while enhancing patient-care outcomes. Future research should also focus on addressing the systemic barriers identified in this study, such as the lack of accessible, safe patient handling training and workplace cultural challenges, to ensure broader applicability and scalability of the intervention. By building on these findings and incorporating lessons learned, we can move closer to developing scalable, sustainable solutions that enhance safety and well-being for community health care workers in diverse settings. Additionally, this feasibility study used brief, adapted TAM items rather than a fully validated instrument tailored to community health care workers. This is not ideal for cross-study comparability, but it was a reasonable, low-burden choice for an early pilot focused on decision signals. Qualitative coding was conducted in Microsoft Excel. While acceptable for small feasibility datasets, Excel lacks features that enhance transparency and credibility. Future studies should consider using validated acceptance measures appropriate to this population, evaluate measurement properties in the target sample, and adopt dedicated qualitative software (eg, NVivo [Lumivero]) with a documented audit trail, multi-coder procedures with agreement checks, and reflexive memoing. Finally, the challenges associated with deploying the app on community health care workers’ devices underscore the need for organizational support and streamlined IT processes. Collaborating with health authorities to address systemic barriers to technology adoption will be essential for scaling the intervention [67]. Future iterations of the app could also incorporate advanced features, such as personalized feedback, gamification elements, and real-time analytics, to enhance user engagement and effectiveness over time.

### Conclusions

To conclude, this single-group feasibility study provides preliminary signals of feasibility and acceptability for the SPH MSIP APP to address unmet safe patient-handling needs among community health care workers. The app was well-received, with high levels of satisfaction and usability reported by participants, suggesting its potential as a practical tool for improving safe patient handling practices. While these findings are promising, they should be interpreted with caution, given the small, self-selected sample and the absence of a control or comparison group; TAM outcomes were used as low-burden, descriptive indicators rather than validated, mechanism-testing measures. Future effectiveness studies should recruit more broadly, use validated acceptance instruments and dedicated qualitative software, and use controlled or randomized designs with longer follow-up to evaluate effects on musculoskeletal injury rates, disability-related absences, and other implementation outcomes.

## Abbreviations

CHS: Community Health Services
mHealth: mobile health
SPH MSIP: Safe Patient Handling Musculoskeletal Injury-Prevention
TAM: Technology Acceptance Model

## Data Availability

The datasets generated or analyzed during this study are available in the Open Science Framework (OSF) repository [68].
